# Head circumference and anthropometric changes and their relation to plexiform and skin neurofibromas in sporadic and familial neurofibromatosis 1 Brazilian adults: a cross-sectional study

**DOI:** 10.1186/s13023-022-02482-8

**Published:** 2022-09-05

**Authors:** Diogo Lisbôa Basto, Gustavo de Souza Vieira, Raquel M. Andrade-Losso, Paula Nascimento Almeida, Vincent M. Riccardi, Rafaela Elvira Rozza-de-Menezes, Karin Soares Cunha

**Affiliations:** 1 Graduate Program in Pathology, School of Medicine, Universidade Federal Fluminense, Niterói, RJ Brazil; 2 Neurofibromatosis National Center, Rio de Janeiro, RJ Brazil; 3 The Neurofibromatosis Institute, Los Angeles, CA USA; 4 Department of Pathology, School of Medicine, Hospital Universitário Antônio Pedro, Universidade Federal Fluminense, Av. Marquês Do Paraná, 303, 4oandar, sala 01. Centro, Niterói, RJ 24033-900 Brazil

**Keywords:** Neurofibromatosis 1, Body height, Body Mass Index, Waist–hip ratio, Neurofibroma

## Abstract

**Background:**

Neurofibromatosis 1 (NF1) is a common autosomal dominant syndrome with complete penetrance and highly variable expressivity. The cutaneous neurofibroma (Cnf) and plexiform neurofibroma (Pnf), café-au-lait spots, and freckle-like lesions are common in NF1, but many other manifestations can occur. We aimed to evaluate head circumference, height, weight, body mass index (BMI), head circumference-to-height ratio (HCHR) and waist–hip ratio (WHR) in adult NF1 Brazilian individuals versus a paired control group and investigate their correlation with the presence of clinically visible Pnfs, and number of “skin neurofibromas” (Snf), which include both cutaneous and subcutaneous neurofibromas.

**Methods:**

A case–control study was conducted with 168 individuals, 84 with NF1 and 84 without NF1, paired by sex and age. Head circumference and anthropometric measurements, Snf quantification, evaluation of clinically visible Pnf and familial inheritance were accessed.

**Results:**

Prevalence of macrocephaly was significantly higher in NF1 women. Height and weight were significantly lower in both males and females with NF1. HCHR was higher in the NF1 group than in the control group for both sexes. BMI was significantly lower in men with NF1. Waist and hip circumferences were significantly reduced in NF compared with the controls, but the mean WHR was significantly lower only in NF1 women. No correlation was found between the Snf and head circumference and anthropometric measurements, sex or family history. The presence and larger size of clinically visible plexiform neurofibromas were associated with normal stature (*p* = 0.037 and *p* = 0.003, respectively).

**Conclusions:**

NF1 individuals have increased prevalence of macrocephaly, short stature, low BMI, and reduced abdominal fat. There is no relation between head circumference and anthropometric data with family history, or neurofibromas.

**Supplementary Information:**

The online version contains supplementary material available at 10.1186/s13023-022-02482-8.

## Introduction

Neurofibromatosis 1 (NF1) is an autosomal dominant tumor predisposing syndrome caused by inherited or de novo mutations in the *NF1* gene, and affects 1:2000 to 1:3000 individuals worldwide [1, 2]. The *NF1* gene encodes neurofibromin, which is a multifunctional protein with a central GAP-related domain (GRD) that negatively regulates the RAS signaling pathway [3, 4].

NF1 presents a complete penetrance and highly variable expressivity [1, 4]. Common clinical manifestations include multiple neurofibromas, café-au-lait spots, freckle-like lesions, Lisch nodules, and bone abnormalities, such as sphenoid dysplasia, bowing of long bones, osteopenia/osteoporosis, macrocephaly, short stature, among others [5–7]. Moreover, there is a lifetime risk of developing malignant peripheral nerve sheath tumor (MPNST) of 8–13% [8, 9].

Neurofibromas are benign neoplasms derived from the peripheral nerve sheath. In NF1, they are ordinarily classified into the localized neurofibroma and Plexiform neurofibroma (Pnf). The localized neurofibroma is small, while the Pnf is a larger tumor caused by extensive involvement along a nerve, usually involving multiple nerve fascicles [10, 11]. Localized neurofibroma often affects the skin (Skin neurofibromas—Snf). They can occur in the dermis (cutaneous neurofibroma; Cnf) or only involve deeper nerve segments in the subcutis (subcutaneous neurofibroma). Neurofibromas usually begin to appear around puberty and increase in size and number during adolescence and pregnancy, suggesting a tissue fat and/or hormonal influences [10, 12–14]. In vitro and in vivo studies have shown the influence of sex steroidal hormones on Cnf and Pnf [15, 16]. Growth hormone (GH) may also influence the development and growth of these tumors [17, 18].

Reduction of lean mass and muscle strength have been reported in NF1 individuals, and are possibly associated with lipid droplets accumulation in skeletal muscle tissue, as observed in *Nf1* heterozygous mice [19]. Moreover, some studies have shown that NF1 persons also present reduced fat mass [20–22]. This feature was recapitulated in *Nf1* heterozygous mice, in which increased density of small adipocytes was found within the visceral and subcutaneous fat depots, suggesting that adipocyte maturation is altered in NF1 [23]. Lipid droplet accumulation was also observed in cells from neurofibromas and MPNSTs from NF1 individuals [24, 25]. These observations suggest that NF1 individuals present a lipid storage phenotype due to metabolic alterations.

Reduction of fat and lean mass can lead to changes in body composition, body measurements, and reduction of weight and body mass index (BMI). In fact, some studies have shown that NF1 individuals present lower weight than the general population and fewer chances of overweight and obesity [26–28]. However, there is no consensus in the literature about BMI in NF1 individuals [20, 21, 26, 29].

The World Health Organization (WHO) indicates that BMI and waist–hip ratio (WHR) are simple methods to identify individuals at increased risk for obesity-related diseases, which include a variety of neoplasm types [30–33]. Obesity has been associated with a state of chronic systemic inflammation, increased serum levels of insulin-like growth factor (IGF-1), and higher levels of estrogens [34–36]. All these alterations contribute to neoplastic promotion and progression [31, 35–37]. Considering the influence of adipocytes in adjacent tissues and the systemic effects of fat mass, it is possible that fat accumulation in overweight and obese NF1 individuals influence the onset and progression of NF1-related manifestations, including the neurofibromas. Despite the lower weight compared to the general populations in some studies, in a recent investigation, we showed that 48% of NF1 individuals were classified as pre-obese and obese based on the BMI measurement and 40% had abdominal obesity based on WHR [24].

There are few studies that investigated head circumference and anthropometric characteristics of NF1 individuals, and most were conducted in children or included children and adults in the same sample. There are even fewer studies in the literature on WHR in these population [7, 20, 21, 24, 26–29, 38–40]. Therefore, we aimed to evaluate head circumference, height, weight, BMI, head circumference-to-height ratio (HCHR) and WHR in adult NF1 Brazilian individuals versus a paired control group and investigate their correlation with the number of Snf and the presence of a visible Pnf.

## Material and methods

### Subjects

This is a cross-sectional study approved by the Ethics Committee (#126/11) of Antônio Pedro University Hospital (HUAP) from Universidade Federal Fluminense (Niterói-Brazil) and a written informed consent was obtained from all participants. A total of 168 post-pubertal Brazilian individuals registered at Antonio Pedro University Hospital were included in this study (84 with NF1 and 84 controls). Antonio Pedro University Hospital serves the Brazilian public health system, known as Unified Health System (SUS) that guarantees equal access to all Health Care Services (HCS). HCS provided by the SUS are usually used by persons of low-income. Therefore, the controls and NF1 participants had the same socioeconomic background. The NF1 diagnosis was based on the diagnostic criteria established by the U.S. National Institutes of Health [5]. The diagnosis was maintained for all individuals even considering the revised criteria published in 2020 [2]. The control group was composed of 84 individuals without NF1, paired by age and sex. Individuals with acute or chronic illness, pregnancy, history of head trauma or surgery, bariatric surgery, and hormone replacement were excluded. Individuals with NF1 with clinically visible Pnf at the reference sites for head circumference and anthropometric measurements were excluded. A large part of the sample of this study (93% of participants) is the same as that used in a previous publication [24].

Age, gender, and skin color were registered for all individuals. For NF1 individuals, the familial history was investigated exhaustively and the data about kyphosis, scoliosis, or lower leg dysplasia were collected based on anamnesis and clinical evaluation. Clinically visible Pnf were diagnosed based on clinical characteristics [41] and registered. Pnf were classified into < 10 cm and ≥ 10 cm of diameter. Snf (cutaneous and subcutaneous) were evaluated and quantified using paper frames, according to Cunha et al. [42], by two independent trained and calibrated examiners. The mean of both counts was considered as the final score for each participant.

### Head circumference and anthropometric measurements

Two trained and calibrated examiners performed the head circumference and anthropometric evaluation. The head circumference was measured from the point just above the eyebrows through the point just above the ear pinning and around the back of the head, according to the Centers for Disease Control and Prevention (CDC) recommendations [43]. Macrocephaly was assumed when values were greater than the 95th percentile, according to age and gender [44].

For the anthropometric measurements, participants were asked to remove their clothes (except for underwear) and shoes. Height and weight were measured using a logical scale attached to a stadiometer (Welmy, model 110 CH, Brazil), and recorded in centimeters and kilograms, respectively. We also accessed the head circumference-to-height ratio (HCHR) following previously published recommendations [45].

Participants were classified with short stature when height was equal to or lower than the 5^th^ percentile from CDC (2011–2014) [43]. BMI was calculated as weight divided by height squared (kg/m^2^), and classified according to criteria set by the WHO [46], in the following categories: Severe underweight (< 16.0); Moderate underweight (16.0–16.9); Mild underweight (17.0–18.49); Normal (18.5–24.9); Overweight (25–29.9); Obesity Class I (moderate) (30–34.9); Obesity Class II (severe) (35–39.9); Obesity Class III (extreme obesity) (> 40).

Waist and hip circumferences were measured using an inextensible plastic measuring tape, suitable for anthropometric measurements. Waist and hip circumference were obtained according to the WHO recommendation: measurement was made at the approximate midpoint between the lower margin of the last palpable rib, and the top of the iliac crest and by positioning the tape around the widest portion of the buttocks, respectively. WHR was calculated and abdominal obesity was defined as WHR ≥ 0.90 for males and ≥ 0.85 for females, according to WHO [47].

### Statistical analysis

Statistical analysis was performed with IBM SPSS® Statistic software (v.20.0, USA). McNemar’s $\chi$^2^ (dichotomous paired data), Friedman’s test (multiple ordinal paired data), and Wilcoxon signed-rank test (continuous paired data) were used for comparison of categorical and continuous variables between NF1 and controls, respectively. Paired *t* test was used to evaluate the age differences between NF1 and controls. Intraclass correlation coefficient (two-way mixed ANOVA model with absolute agreement) and a paired *t* test were used to compare the means of Snf quantification between the two examiners. Mann–Whitney and Pearson Chi-square tests were applied to evaluate variables within the NF1 sample and correlate with Snf and Pnf, respectively. The odds ratios (ORs) with 95% confidence interval (CI) were also applied to estimate explanatory variables. Post Hoc procedures were used for interpreting significant p-values from contingency table test results using the standardized residual method, if necessary [48]. Multiple linear and binary logistic regressions using the "hierarchical method" (Block [1]: scoliosis and/or kyphosis and long bone dysplasia; Block [2]: scoliosis and/or kyphosis, long bone dysplasia, plexiform neurofibroma and the number of skin neurofibromas) were used to assess the impact of these independent variables on alterations in head circumference and anthropometric findings in NF1 group. Quantitative variables are either reported as percentage, mean ± standard deviation (SD), median, first and third quartile or converted to categorical variables. All tests were two-tailed and *p* values < 0.05 were considered statistically significant.

## Results

Mean age for the NF1 group was 43.95 ± 13.8 years and for the control group 44.2 ± 13.9 years. There was no significant difference in age between both groups (*p* = 0.225, Paired *t* test). Of the 84 participants of each group (NF1 and control), 55 (65.5%) were women and 29 (34.5%) were men. Thirty-three (39.3%) participants were white, and fifty-one (60.7%) were black in the NF1 group. Forty-two (50.0%) were white, and 42 (50.0%) were black in the control group.

**Fig. 1:**
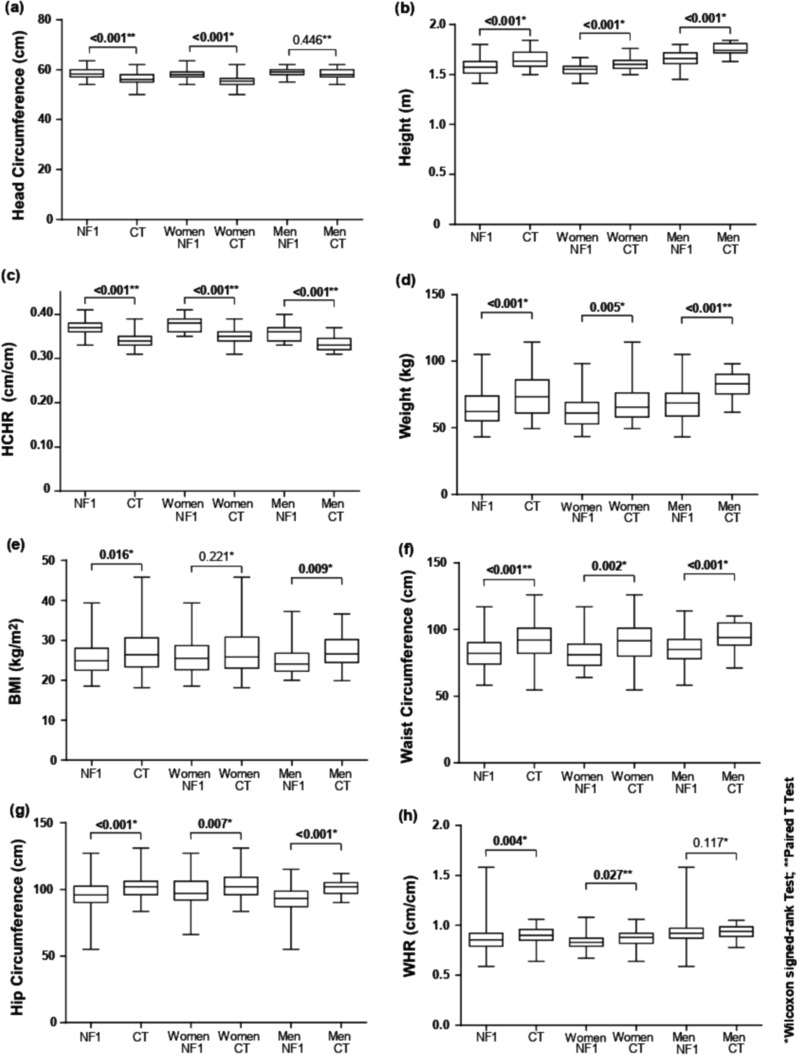
Box plot graphics of the comparison of anthropometric data from neurofibromatosis 1 and control groups. **a** Comparison between the neurofibromatosis 1 and control groups, with a gender split as well, for the head circumference (cm) variable. **b** Comparison between the neurofibromatosis 1 and control groups, with a gender split as well, for the height (m) variable. **c** Comparison between the neurofibromatosis 1 and control groups, with a gender split as well, for the head circumference height ratio (cm/m) variable. **d** Comparison between the neurofibromatosis 1 and control groups, with a gender split as well, for the weight (kg) variable. **e** Comparison between the neurofibromatosis 1 and control groups, with a gender split as well, for the body mass index (kg/m^2^) hit circumference (cm) variable. **f** Comparison between the neurofibromatosis 1 and control groups, with a gender split as well, for the waist circumference (cm) variable. **g** Comparison between the neurofibromatosis 1 and control groups, with a gender split as well, for the hip circumference (cm) variable. **h** Comparison between the neurofibromatosis 1 and control groups, with a gender split as well, for the waist/hip ratio (cm/cm) variable

Data about head circumference are presented in Fig. 1a. NF1 individuals had head circumference (58.43 cm ± 1.93) larger than controls (56.43 cm ± 2.35) (Fig. 1a). Evaluating sexes separately, this difference was maintained only in the female group. Macrocephaly was significantly more prevalent in women with NF1 (n = 39; 70.9%) than in control women (n = 16; 29.1%) (Table 1).

**Table 1:** Comparison of head circumference and anthropometric classifications between neurofibromatosis 1 and control groups

	NF1 (n = 84/100%)	CT (n = 84/100%)	*p*-value (NF1-CT)	NF1 Women (n = 55/100%)	CT Women (n = 55/100%)	*p*-value Woman (NF1-CT)	NF1 Men (n = 29/100%)	CT Men (n = 29/100%)	*p*-value Men (NF1-CT)
Macrocephaly									
Yes	49 (58.3%)	15 (17.9%)	** < 0.001**^**a**^	39 (70.9%)	7 (12.7%)	** < 0.001**^**a**^	10 (34.5%)	8 (27.6%)	0.791^a^
No	35 (41.7%)	69 (82.1%)		16 (29.1%)	48 (87.3%)		19 (65.5%)	21 (72.4%)	
Stature									
Normal	55 (65.5%)	80 (95.2%)	** < 0.001**^**a**^	39 (70.9%)	52 (94.5%)	**0.002**^**a**^	16 (55.2%)	28 (96.6%)	** < 0.001**^**a**^
Short Stature	29 (34.5%)	4 (4.8%)		16 (29.1%)	3 (5.5%)		13 (44.8%)	1 (3.4%)	
BMI (kg/m^2^)									
Severe underweight	–	–	**0.036**^**b**^	**–**	**–**	0.317^b^	**–**	**–**	**0.041**^**b**^
Moderate underweight	–	–		–	–		–	–	
Mild underweight	–	1 (1.2%)		–	1 (1.8%)		–	–	
Normal	42 (50.0%)	35 (41.7%)		25 (45.5%)	26 (47.3%)		17 (58.6%)	9 (31%)	
Overweight	30 (35.7%)	23 (27.4%)		21 (38.2%)	11 (20.0%)		9 (31.0%)	12 (41.4%)	
Obesity Class I	9 (10.7%)	**19 (22.6%)**		7 (12.7%)	12 (21.8%)		2 (6.9%)	7 (24.1%)	
Obesity Class II	3 (3.6%)	**4 (4.8%)**		2 (3.6%)	3 (5.5%)		1 (3.4%)	1 (3.4%)	
Obesity Class III	–	**2 (2.4%)**		–	2 (3.6%)		–	–	
WHR									
Normal WHR score	49 (58.3%)	30 (35.7%)	**0.004**^**a**^	36 (65.5%)	20 (36.4%)	**0.004**^**a**^	13 (44.8%)	8 (27.6%)	0.581^a^
High WHR score	35 (41.7%)	54 (64.3%)		19 (34.5%)	35 (63.6%)		16 (55.2%)	21 (72.4%)	

Significant *p* values (< 0.05) are in bold

NF1 = Neurofibromatosis 1 group; CT = Control group; BMI = Body mass index; WHR = Waist/hip ratio; ^a^McNemar’s $\chi$^2^ test; ^b^Friedman’s test

Data about height are presented in Fig. 1b. Mean height was significantly lower in the NF1 group, at 165.0 ± 8 cm in men and 154.0 ± 6 cm in women. In the control group, the mean height was 174.0 ± 6 cm in men and 160.0 ± 5 cm in women. Twenty-nine (34.5%) individuals from the NF1 group had short stature versus four (4.8%) control individuals, demonstrating a significantly higher frequency of short stature in NF1 than in controls for both men and women (Table 1).

The results for HCHR are shown in Fig. 1c. The HCHR was higher in the NF1 group than in the control group (0.37 ± 0.019 versus 0.34 ± 0.015, p < 0.001). When sexes were compared, a higher ratio was noted in NF1 versus control group for both sexes (Fig. 1c). Linear regression is shown in Additional file 1: Fig. S1.

**Fig. 2:**
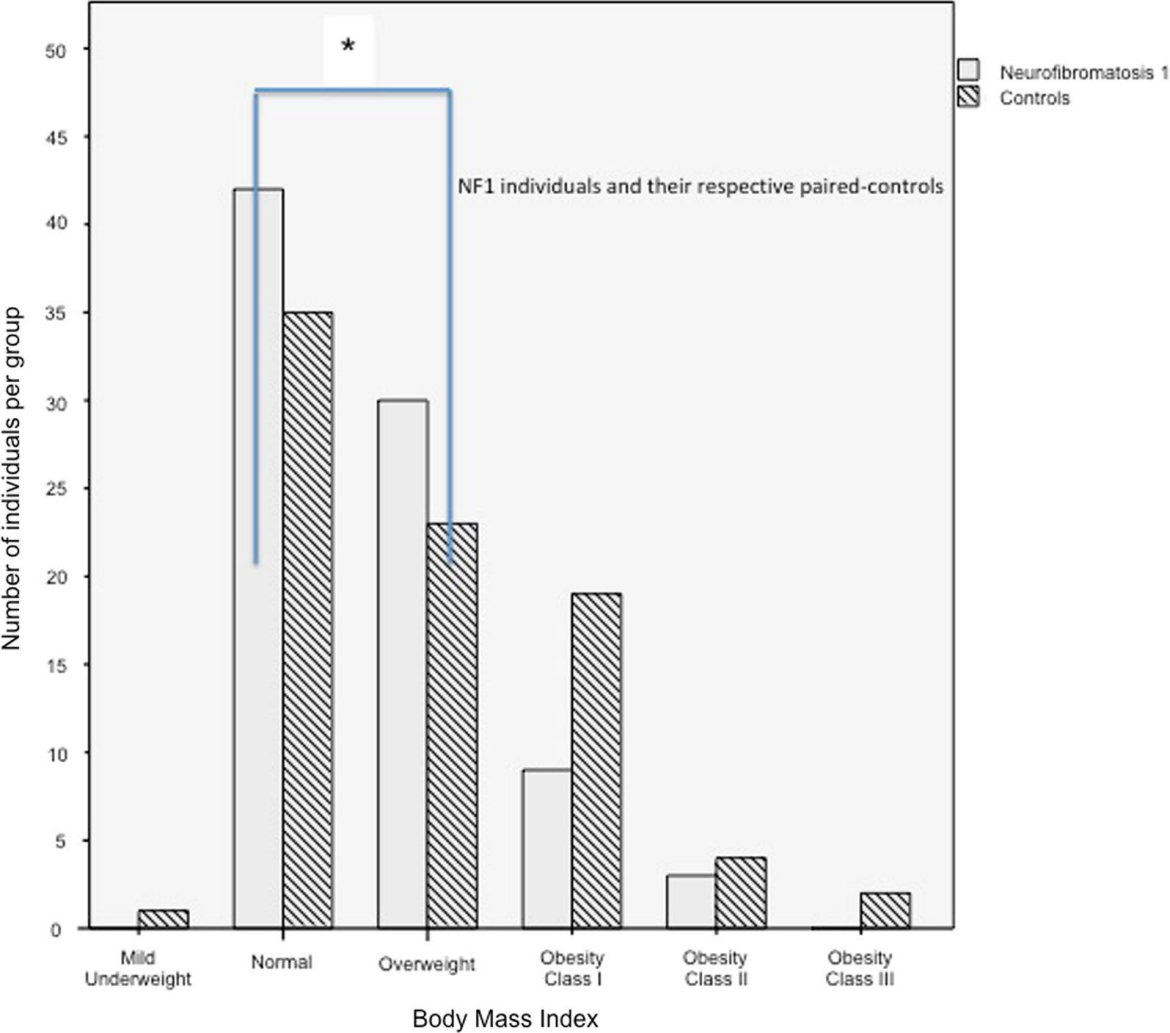
Post-Hoc difference analysis between neurofibromatosis 1 and control groups for body mass index classificatory categories

Both NF1 men and women had significantly lower weight than the controls (Fig. 1d). Table 1 and (Fig. 2) shows the percentage of BMI classification according to groups. In addition, NF1 participants had lower BMI than the controls (25.6 kg/m^2^ ± 4.3 versus 27.1 kg/m^2^ ± 5.3, *p* = 0.016). The post-hoc analysis of the prevalence of BMI categories is presented in Fig. 2 and Additional file 2: Fig. S2, which shows the specific differences between the prevalence of normal BMI in NF1 while the paired controls were overweight. Male NF1 individuals had significantly lower mean BMI than male controls (25.6 kg/m^2^ ± 4.3 vs. 27.1 kg/m^2^ ± 5.3, *p* = 0.009) (Fig. 1e). No statistically significant difference in BMI was observed between NF1 women and control women.

Waist and hip circumferences and WHR were statistically significant reduced in the NF1 group compared with controls (WHR: 0.87 ± 0.12 versus 0.90 ± 0.08, *p* = 0.004; Fig. 1f, g and h). Nevertheless, the mean WHR was significantly lower only in NF1 women compared with female controls (0.84 ± 0.7 vs. 0.87 ± 0.09, *p* = 0.027) (Fig. 1h).

The Snf count was obtained from 81 participants. Three individuals did not want to be submitted to the photographs. The intraclass correlation analysis for the Snf count showed a strong concordance between the two examiners (average of 0.996 with 0.994–0.997 95% confidence interval, two-way mixed ANOVA model; *p* < 0.001). The Snf number varied from none to 837.50 tumors per 300 cm^2^ of skin (first quartile = 49.75; median = 158.00; third quartile = 296.25). Thirty (35.7%) individuals had at least one clinically visible Pnf (head and neck: 9.5%; trunk: 4.8%; upper and lower limbs: 6% and 11.9%, respectively; hip: 3.6%). The Snf number showed no statistical association with sex, family history of NF1, presence of clinically visible Pnf, or any of the categorical anthropometric variables (Table 2). Thirty participants had clinically visible Pnfs and 67% had a size ≥ 10 cm. Clinically visible Pnf was four times more frequent in individuals of normal stature (24.80% individuals with normal stature status versus 6.20% individuals with short stature; *p* = 0.037; Pearson chi-square test, Table 3), the majority of these individuals (85%) also had larger Pnfs (*p* = 0.03; McNemar’s $\chi$^2^ test).

**Table 2:** Relation of the skin neurofibroma count with sex, familial history, plexiform neurofibroma and anthropometric classifications in individuals with neurofibromatosis 1

NF1	Skin neurofibromas			
	n	Median	Mean ± Standard Deviation	*p*-value^a^
Sex	n = 81 (100%)			
Men	27 (33.3%)	134.00	185.37 ± 180.92	0.656
Woman	54 (66.7%)	158.00	209.87 ± 196.90	
Familial History	n = 80 (100%)			
Positive	51 (63.7%)	134.00	207.13 ± 206.48	0.940
Negative	29 (36.3%)	158.00	180.36 ± 152.75	
Plexiform neurofibroma	n = 81 (100%)			
Yes	30 (37.0%)	96.25	197.13 ± 221.56	0.431
No	51 (63.0%)	161.00	204.39 ± 172.74	
Macrocephaly	n = 81 (100%)			
Yes	49 (60.5%)	134.00	202.01 ± 195.28	0.908
No	32 (39.5%)	162.75	201.23 ± 187.19	
Stature	n = 81 (100%)			
Normal	27 (33.3%)	113.00	188.62 ± 187.81	0.316
Short Stature	54 (66.7%)	213.00	227.87 ± 198.01	
BMI	n = 81 (100%)			
Normal	41 (50.6%)	191.50	200.74 ± 161.76	0.673
Overweight	29 (35.8%)	146.00	220.16 ± 234.19	
Obese	11 (13.6%)	93.00	156.64 ± 173.80	
WHR	n = 81 (100%)			
Normal WHR score	48 (59.3%)	152.00	197.57 ± 194.28	0.818
High WHR score	33 (40.7%)	161.00	207.71 ± 188.79	

NF1: Neurofibromatosis 1; BMI: Body mass index; WHR: Waist/hip ratio; ^a^Mann-Whitney test

**Table 3:** Relation of the plexiform neurofibroma with sex and anthropometric classifications in individuals with neurofibromatosis 1

NF1 n = 84 (100%)	Plexiform neurofibroma		
	Present n = 30 (35.7%)	Absent n = 54 (64.3%)	*p* value
Sex			
Woman	21 (70.0%)	34 (63.0%)	0.516^a^
Men	9 (30.0%)	20 (37.0%)	
Macrocephaly			
Yes	17 (56.7%)	32 (59.3%)	0.817^a^
No	13 (43.3%)	22 (40.7%)	
Stature			
Normal	24 (80.0%)	31 (57.4%)	**0.037**^a^
Short Stature	6 (20.0%)	23 (42.6%)	
BMI			
Normal	17 (56.7%)	25 (46.3%)	0.671^b^
Overweight	9 (30.0%)	21 (38.9%)	
Obese	4 (13.3%)	8 (14.8%)	
WHR			
Normal WHR score	18 (60.0%)	31 (57.4%)	0.817^a^
High WHR score	12 (40.0%)	23 (42.6%)	

Significant *p* values (< 0.05) are in bold

NF1: Neurofibromatosis 1; BMI = Body mass index; WHR = Waist/hip ratio; ^a^Pearson Chi-square test; ^b^Fisher’s Exact test

Fifty-three (63.1%) of NF1 individuals had one or more relatives with NF1, and for one individual this information was missing due to adoption. There was no relationship between the inherited or sporadic NF1 with anthropometric measurements (Table 4).

**Table 4:** Relation of familial history with plexiform neurofibromas and anthropometric classifications in individuals with neurofibromatosis 1

NF1 n = 83 (100%)	Familial History		
	Positive n = 53 (63.9%)	Negative n = 30 (36.1%)	*p* value
Sex			
Men	20 (37.7%)	9 (30%)	0.478^a^
Woman	33 (62.3%)	21 (70%)	
Plexiform neurofibroma			
Present	21 (39.6%)	9 (30%)	0.381^a^
Absent	32 (60.4%)	21 (70%)	
Macrocephaly			
Yes	32 (60.4%)	16 (53.3%)	0.532^a^
No	21 (39.6%)	14 (46.7%)	
Stature			
Normal	32 (60.4%)	22 (73.3%)	0.234^a^
Short Stature	21 (39.6%)	8 (26.7%)	
BMI			
Normal	26 (49.1%)	16 (51.6%)	**0.029**^**b**^
Overweight	23 (43.4%)	7 (23.3%)	
Obese	4 (7.5%)	8 (26.7%)	
WHR			
Normal WHR score	22 (41.5%)	13 (43.3%)	0.872^a^
High WHR score	31 (58.5%)	17 (56.7%)	

Significant *p* values (< 0.05) are in bold

NF1: Neurofibromatosis 1; BMI: Body mass index; WHR: Waist/hip ratio; ^a^Pearson Chi-square test; ^b^Fisher’s Exact test

Hierarchical regression analysis was performed to assess whether scoliosis and/or kyphosis (present in 46% of NF1 individuals), long bone dysplasia (present in 7.6% of NF1 individuals), plexiform neurofibroma (present in 37% of NF1 individuals) and the number of skin neurofibromas had an impact on head circumference and anthropometric measurements. The results are shown in Additional file 3: Fig. S3 and indicate that only plexiform neurofibroma had an impact, as an independent variable, on height. Pnf explained an additional 11.7% (Block [1] R^2^ = 0.003; Block [2] R^2^ = 0.12; R^2^[1] – R^2^[2] or 0.003–0.12 = − 0.117) of the variance in the height of NF1, which was statistically significant (*p* = 0.032).

## Discussion

Our study presents an investigation of the head circumference and anthropometric characteristics of Brazilian adults with NF1 and is a novel assessment of a possible association between the alteration of these measurements and the presence of neurofibromas.

Macrocephaly is common in NF1, occurring in 38–75% in previous studies [39, 49, 50]. However, most of these investigations evaluated children and adolescents [51–54]. In the present study with an adult population, macrocephaly occurred in 58.3% of NF1 individuals. Nevertheless, evaluating sexes separately, the prevalence of macrocephaly was higher only in NF1 women compared with the controls [39, 49, 50]. The few studies that assessed the head circumference measurements in NF1 separately in males and females, showed that macrocephaly was more frequent in men, different from our results [40, 50, 55, 56].

Some studies support that there is a correlation between head circumference and height and suggest that absolute measurements of head size without regard to stature are inadequate for demonstrating clinically significant macrocephaly [39, 45]. Assessment of head circumference with respect to height is especially important in some growth disorders, including NF1, in which discordance between head circumference and height is a characteristic feature [39]. Therefore, we evaluated the HCHR. Our results were similar to previous studies showing that NF1 individuals show a greater HCHR than healthy individuals [39, 40, 43]. When evaluating sexes separately, Souza et al. [40] observed that NF1 males had higher HCHR compared to females, while in our study, we showed a higher HCHR in females compared to males (0.37 ± 0.016 and 0.35 ± 0.015, respectively; Student’s *T* test, *p* < 0.001).

In NF1, macrocephaly has been associated with a large brain volume due to the enlargement of white and grey matter [57, 58]. A larger head circumference was already pointed as a clinical indicator for the presence of optic pathway gliomas in NF1 [54], however, this is still debated [59].

A significantly higher frequency of short stature was observed in the NF1 group (34.5%) compared with the control group (4.8%) for both men and women. This result is consistent with previous literature data that report short stature in adults with NF1, with percentages that vary between 27 and 60%. [40, 60] Short stature in NF1 may be explained by an abnormal hypothalamic-pituitary axis function, that is thought to play a causative role in the growth defect [61, 62]. Hegedus et al. [63] demonstrated that neurofibromin regulates the function of the hypothalamic-pituitary axis by modulating intra-cellular cAMP levels. Neurofibromin loss in the brain of murine models leads to decreased GH and IGF-1 levels [63]. Short stature in NF1 may also be associated with bone defects, such as scoliosis or congenital tibial dysplasia [61, 62], but this association was not observed in the present study.

The presence of macrocephaly associated with short stature shows that NF1 affects differently the growth of long bones and the development of the cranium bones [64].

In our sample, although both men and women with NF1 had significant reduced weight, BMI was lower only in men with NF1 compared with the control males. There is no consensus in the literature about the BMI of NF1 individuals. In a Brazilian study, there was no difference in BMI between NF1 individuals and controls [20]. In a study with Jewish military recruits with NF1 in Israel, BMI, although lower in NF1 individuals, was not significantly different in comparison to the controls [30]. On the other hand, in another study with an Italian sample, individuals with NF1 had lower BMI than the control group [7]. Nevertheless, sexes were not evaluated separetetly [7]. In two studies with a Japanese sample, Koga et al. [26, 29] observed significantly lower BMI only in NF1 men compared with the controls, similarly to the results found in our study. On the other hand, although in our Brazilian sample, NF1 individuals had significantly lower waist and hip circumferences in comparison to the controls for both sexes, WHR was significantly reduced only in NF1 women compared with the control females. Other studies did not find significant differences in waist circumferences in NF1 individuals compared with the controls [7, 21]. More studies are needed to better understand the body composition in NF1 and to determine if this syndrome affects men and women in different ways in their head circumference and anthropometric characteristics.

Previous studies have shown that NF1 individuals present reduced physical fitness and insufficient dietary intake of nutrients that could be related to low BMI, WC and WHR found in this population. Nevertheless, emerging evidence suggests that dysregulation of Ras signaling in NF1 causes cellular and organismal metabolic changes that could explain the body composition alterations found in this and previous studies. For instance, increased insulin sensitivity, reduced serum glucose levels, hypometabolism of cerebral glucose, and low chances of occurrence of type 2 diabetes mellitus have been found in NF1 individuals [65, 66]. As described earlier in this paper, increased cellular lipid storage is common in tissues from NF1 individuals [24, 25]. Moreover, NF1 individuals, particularly women, present increased resting energy expenditure [21]. Altogether, these observations suggest that neurofibromin haploinsufficiency affects uptake, storage, and expenditure of energy [23, 30, 67]. In a recent study, Botero et al [68] showed that neurofibromin regulates metabolic homeostasis in *Drosophila*, increasing the metabolic rate, feeding, and energy homeostasis via actions on a central neuronal circuit.

Since adipocytes are ubiquitous and provide local and systemic biochemical signals to surrounding cells, regulating cell proliferation, migration, and differentiation [23], we investigated if the body composition of NF1 individuals exerts an impact on the developing of neurofibromas. Weight, BMI, WC, HC and WHR were not correlated with these lesions. Nevertheless, interestingly, previous studies showed that NF1-associated neurofibromas present neoplastic Schwann cells with lipid droplets accumulation, express leptin and have higher chances of having intermingled adipocytes than non NF1-associated neurofibromas [24, 69–71]. Lipid droplets accumulation and overexpression of fatty acid synthesis (FASN) were also found in MPNSTs [25]. In a previous study, we did not find a correlation between weight, WC, HC and WHR with the presence of adipocytes in neurofibromas [24]. Therefore, metabolic alterations in neurofibroma cells may be independent from the body fat accumulation in NF1 individuals. The influence of systemic metabolic alterations on timing and development of neurofibromas and other NF1-associated neoplasms need to be more explored in future studies.

A statistically significant relationship between the presence of clinically visible Pnf and normal height was observed in the present study. Moreover, larger Pnf were present in the tallest individuals with NF1 in our sample. A previous investigation showed that NF1 microdeletion patients usually are taller than other NF1 patients [72] and it is known that microdeletion NF1 patients have more chances of developing a Pnf [73]. We did not investigate the germline *NF1* mutation type of these participants. Maybe part of our sample that had normal stature and clinically visible Pnf is composed of *NF1* microdeletion individuals. However, the prevalence of microdeletion in patients with NF1 is about 5–10% [74], which is much lower than the prevalence of 28.6% of individuals with normal height and clinically visible Pnf present in our sample. Future studies comparing the height of NF1 individuals with and without Pnf are needed to understand this correlation.

One important limitation of this study is the limited sample size. Future studies with other populations and large samples are needed to better understand the anthropometric and head circumference alterations in NF1, if they are different for men and women**,** and whether body composition could affect the timing and development of NF1-associated neurofibromas and other neoplasms.

## Conclusion

NF1 individuals have increased prevalence of macrocephaly, short stature, low BMI, and reduced abdominal fat. Some changes in body composition are different in men and women with NF1. There is no correlation between head circumference and anthropometric data, family history, or neurofibromas.

## Supplementary Information

**Additional file 1**. **Fig. S1** Linear regression model of the relation of head circumference and weight data from NF1 and control individuals. (**a**) Relation observed in the neurofibromatosis 1 group, (**b**) control group, (**c**) women in the neurofibromatosis 1 group, (**d**) women in the control group, (**e**) men in the neurofibromatosis 1 group, (**f**) and the men in the control group.

**Additional file 2**. **Fig. S2** Calculation of cell chi-square, standardized residuals, relative contribution and cell *p* value of Body Mass Index according to the contingency table for neurofibromatosis 1 and control groups.

**Additional file 3**. **Fig. S3** Results of the hierarchical binomial logistic or multiple linear regression analysis about head circumference and anthropometric changes after adjusting the body impacting alterations in neurofibromatosis 1.

## Data Availability

The datasets used and/or analyzed during the current study are available from the corresponding author on reasonable request.

## Abbreviations

NF1: Neurofibromatosis 1
GAP: GTPase-activating protein
GRD: GAP-related domain
RAS: Rat sarcoma virus
MPNST: Malignant peripheral nerve sheath tumor
Pnf: Plexiform neurofibroma
Snf: Skin neurofibroma
Cnf: Cutaneous neurofibroma
GH: Growth hormone
BMI: Body mass index
WHO: World Health Organization
WHR: Waist–hip ratio
IGF-1: Insulin growth factor 1
HCHR: Head circumference-to-height ratio
CDC: Centers for Disease Control and Prevention
USA: United States of America
OR: Odds ratio
CI: Confidence interval
SD: Standard deviation
cAMP: Cyclic adenosine monophosphate
