# Supplementary material for: Head circumference and anthropometric changes and their relation to plexiform and skin neurofibromas in sporadic and familial neurofibromatosis 1 Brazilian adults: a cross-sectional study
Source: Orphanet J Rare Dis. 2022 Sep 5;17:341. doi: 10.1186/s13023-022-02482-8 (PMC9446792; doi:10.1186/s13023-022-02482-8)

**Additional file 1**– Linear regression model of the relation of head circumference and weight data from NF1 and control individuals.


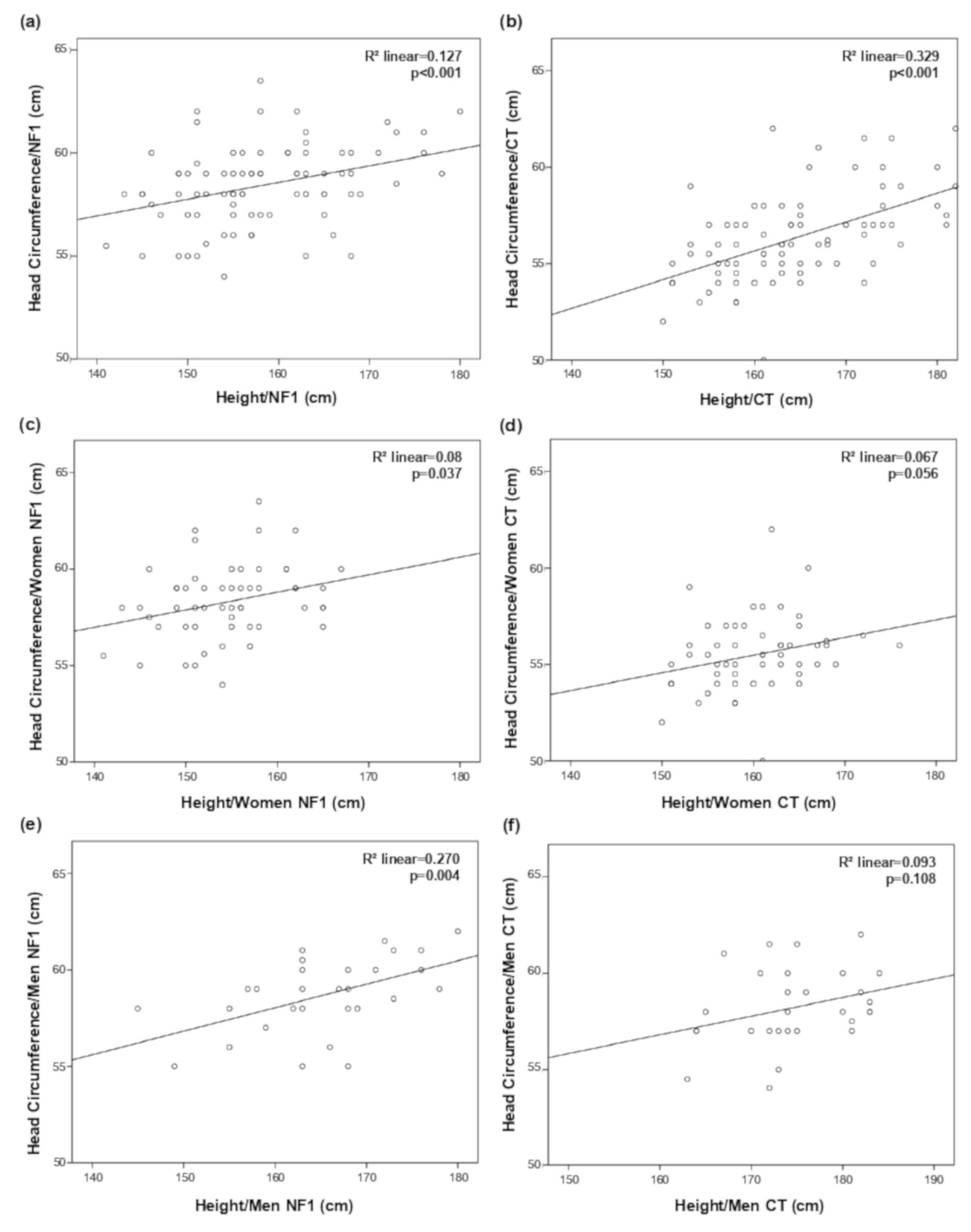

Supplement: Supplementary file 1 — Additional file 1. Fig. S1 Linear regression model of the relation of head circumference and weight data from NF1 and control individuals. (a) Relation observed in the neurofibromatosis 1 group, (b) control group, (c) women in the neurofibromatosis 1 group, (d) women in the control group, (e) men in the neurofibromatosis 1 group, (f) and the men in the control group. [file 13023_2022_2482_MOESM1_ESM.docx]
