# Supplementary material for: Head circumference and anthropometric changes and their relation to plexiform and skin neurofibromas in sporadic and familial neurofibromatosis 1 Brazilian adults: a cross-sectional study
Source: Orphanet J Rare Dis. 2022 Sep 5;17:341. doi: 10.1186/s13023-022-02482-8 (PMC9446792; doi:10.1186/s13023-022-02482-8)
Supplement: Supplementary file 2 — Additional file 2. Fig. S2 Calculation of cell chi-square, standardized residuals, relative contribution and cell p value of Body Mass Index according to the contingency table for neurofibromatosis 1 and control groups. [file 13023_2022_2482_MOESM2_ESM.docx]

**Additional file 2** - Calculation of cell chi-square, standardized residuals, relative contribution and cell p-value of Body Mass Index according to the contingency table for neurofibromatosis 1 and control groups

| Cell | Frequency | Cell chi-square | Standardized Residuals | Relative (%) contribution^1^ | *p-value*^2^ |
| --- | --- | --- | --- | --- | --- |
| R_1_C_1_ | 1 | 0.50 | 1.0 | 3.8110 | 0.32 |
| R_1_C_2_ | 0 | 0.40 | -0.7 | 3.0488 | 0.48 |
| R_1_C_3_ | 0 | 0.10 | -0.3 | 0.7622 | 0.76 |
| R_1_C_4_ | 0 | 0.00 | -0.2 | 0 | 0.84 |
| R_2_C_1_ | 16 | 0.12 | -0.7 | 0.9146 | 0.48 |
| R_2_C_2_ | 13 | 0.02 | 0.2 | 0.1524 | 0.84 |
| R_2_C_3_ | 4 | 0.01 | 0.2 | 0.0762 | 0.84 |
| R_2_C_4_ | 2 | 0.38 | 0.9 | 2.8963 | 0.37 |
| R_3_C_1_ | 16 | 1.77 | 2.2 | **13.4909** | **0.002** |
| R_3_C_2_ | 5 | 1.25 | -1.6 | 9.5274 | 0.10 |
| R_3_C_3_ | 1 | 0.9 | -1.2 | 6.8598 | 0.23 |
| R_3_C_4_ | 1 | 0.05 | 0.2 | 0.3811 | 0.84 |
| R_4_C_1_ | 6 | 1.29 | -1.8 | 9.8323 | 0.07 |
| R_4_C_2_ | 10 | 1.50 | 1.7 | 11.4329 | 0.08 |
| R_4_C_3_ | 3 | 0.50 | 0.8 | 3.8110 | 0.42 |
| R_4_C_4_ | 0 | 0.70 | -1.0 | 5.3354 | 0.31 |
| R_5_C_1_ | 1 | 0.50 | -1.0 | 3.8110 | 0.31 |
| R_5_C_2_ | 2 | 0.25 | 0.6 | 1.9055 | 0.54 |
| R_5_C_3_ | 1 | 0.90 | 0.9 | 6.8598 | 0.36 |
| R_5_C_4_ | 0 | 0.10 | -0.4 | 0.7622 | 0.68 |
| R_6_C_1_ | 2 | 1.00 | 1.4 | 7.6220 | 0.16 |
| R_6_C_2_ | 0 | 0.70 | -1.1 | 5.3354 | 0.27 |
| R_6_C_3_ | 0 | 0.10 | -0.5 | 0.7622 | 0.61 |
| R_6_C_4_ | 0 | 0.08 | -0.3 | 0.6098 | 0.76 |
| Total χ^2^_(15)_ |  | **=13.12** |  |  |  |
| Total |  |  |  | **=100%** |  |

^1^ The relative contribution method is computed by dividing each cell chi-square by the total chi-square value (%=x100); ^2^ The two-tailed critical value of *z* at the α type error adjusted (α_adj_) of 0.0043 (Sidak, 1967; α_adj_ = 1 – (1- α)^1/t^, the *t* equals the number of tests; α_adj_ = 1-(1-0.05)^1/12^). The p-value was calculated for each cell chi-square using SIG.CHISQ tool (IBM SPSS® Statistics, version 20.0). Legend: R=row (controls), R_1_: mild underweight, R_2_: normal, R_3_: overweight, R_4_: Obesity class I, R_5_: Obesity class II, R_6_: Obesity class III; C=column (neurofibromatosis 1), C_1_: normal, C_2_: overweight, C_3_: Obesity class I, C_4_: Obesity class II.
