# Supplementary material for: Head circumference and anthropometric changes and their relation to plexiform and skin neurofibromas in sporadic and familial neurofibromatosis 1 Brazilian adults: a cross-sectional study
Source: Orphanet J Rare Dis. 2022 Sep 5;17:341. doi: 10.1186/s13023-022-02482-8 (PMC9446792; doi:10.1186/s13023-022-02482-8)
Supplement: Supplementary file 3 — Additional file 3. Fig. S3 Results of the hierarchical binomial logistic or multiple linear regression analysis about head circumference and anthropometric changes after adjusting the body impacting alterations in neurofibromatosis 1. [file 13023_2022_2482_MOESM3_ESM.docx]

**Additional file 3.** Results of the hierarchical binomial logistic or multiple linear regression analysis about head circumference and anthropometric changes after adjusting the body impacting alterations in neurofibromatosis 1

| *Dependent variable*: Macrocephaly (binomial)  ***R^2^ (Nagelkerke)*=0.032; *χ^2^*=1.783;df=4*; p-*value=0.776**  *Independent variables* | Exp (β) | 95% I.C. | *p*-value |
| --- | --- | --- | --- |
| Kyphosis or scoliosis | 1.51 | 0.5-3.9 | 0.399 |
| Alterations of the long bones | 0.32 | 0.03-3.2 | 0.337 |
| Plexiform neurofibroma(s) | 1.17 | 0.4-3.1 | 0.755 |
| Skin neurofibromas | 1.00 | 0.9-1.0 | 0.921 |
| *Dependent variable*: Stature (binomial)  ***R^2^ (Nagelkerke)*=0.120;*χ^2^*=6.710;df=4*; p-*value=0.152**  *Independent variables* | Exp (β) | 95% I.C. | *p*-value |
| Kyphosis or scoliosis | 0.953 | 0.34-2.6 | 0.927 |
| Alterations of the long bones | 0.516 | 0.06-4.2 | 0.536 |
| Plexiform neurofibroma(s) | 3.593 | 1.2-11.6 | **0.032** |
| Skin neurofibromas | 0.998 | 0.98-1.0 | 0.209 |
| *Dependent variable*: Weight (linear)^a^  ***R^2^*=0.050;*F*=0.917; *p-*value=0.459**  *Independent variables* | Exp (β) | 95% I.C. | *p*-value |
| Kyphosis or scoliosis | 2.192 | -3.9 - 9.0 | 0.476 |
| Alterations of the long bones | 0.730 | -11.4 - 12 | 0.904 |
| Plexiform neurofibroma(s) | 0.442 | -5.9 - 6.7 | 0.889 |
| Skin neurofibromas | -0.013 | -0.02 - 0.003 | 0.108 |
| *Dependent variable*: Body mass index (linear)^a^  ***R^2^*=0.038;*F*=0.684; *p-*value=0.606**  *Independent variables* | Exp (β) | 95% I.C. | *p*-value |
| Kyphosis or scoliosis | 0.119 | -1.03 - 3.09 | 0.324 |
| Alterations of the long bones | -0.093 | -5.68 - 2.48 | 0.437 |
| Plexiform neurofibroma(s) | 0.097 | -1.25 - 2.98 | 0.419 |
| Skin neurofibromas | -0.076 | -0.007 - 0.004 | 0.526 |
| *Dependent variable*: Waist-hip ratio (linear)^a^  ***R^2^*=0.025;*F*=0.441; *p-*value=0.778**  *Independent variables* | Exp (β) | 95% I.C. | *p*-value |
| Kyphosis or scoliosis | -0.058 | -0.07 - 0.04 | 0.632 |
| Alterations of the long bones | 0.087 | -0.07 - 0.15 | 0.473 |
| Plexiform neurofibroma(s) | -0.129 | -0.09 - 0.02 | 0.286 |
| Skin neurofibromas | -0.041 | -0.00017-0.00011 | 0.735 |

^a^The collinearity diagnosis using variance inflation factor (VIF) and tolerance showed satisfactory results about independent variables inserted in the model.
